# Common Hydrogen Bond Interactions in Diverse Phosphoryl Transfer Active Sites

**DOI:** 10.1371/journal.pone.0108310

**Published:** 2014-09-19

**Authors:** Jean C. Summerton, Gregory M. Martin, Jeffrey D. Evanseck, Michael S. Chapman

**Affiliations:** 1 Department of Biochemistry and Molecular Biology, School of Medicine, Oregon Health and Science University, Portland, Oregon, United States of America; 2 Center for Computational Sciences and the Department of Chemistry and Biochemistry, Duquesne University, Pittsburgh, Pennsylvania, United States of America; Oak Ridge National Laboratory, United States of America

## Abstract

Phosphoryl transfer reactions figure prominently in energy metabolism, signaling, transport and motility. Prior detailed studies of selected systems have highlighted mechanistic features that distinguish different phosphoryl transfer enzymes. Here, a top-down approach is developed for comparing statistically the active site configurations between populations of diverse structures in the Protein Data Bank, and it reveals patterns of hydrogen bonding that transcend enzyme families. Through analysis of large samples of structures, insights are drawn at a level of detail exceeding the experimental precision of an individual structure. In phosphagen kinases, for example, hydrogen bonds with the O_3β_ of the nucleotide substrate are revealed as analogous to those in unrelated G proteins. In G proteins and other enzymes, interactions with O_3β_ have been understood in terms of electrostatic favoring of the transition state. Ground state quantum mechanical calculations on model compounds show that the active site interactions highlighted in our database analysis can affect substrate phosphate charge and bond length, in ways that are consistent with prior experimental observations, by modulating hyperconjugative orbital interactions that weaken the scissile bond. Testing experimentally the inference about the importance of O_3β_ interactions in phosphagen kinases, mutation of arginine kinase Arg_280_ decreases k_cat_, as predicted, with little impact upon K_M_.

## Introduction

Enzymes that catalyze the transfer of a phosphate from ATP are widespread in biology. Free energy liberated in hydrolysis of phosphoandyride bonds, such as those in ATP, is essential for cellular energy metabolism, motility and the generation of transmembrane potentials. Much has been learned about enzyme-catalyzed phosphoryl transfer through the detailed study of individual enzymes. The most frequently cited mechanisms include; a) precise positioning of substrates for phosphate transfer; b) base-assisted activation of the nucleophile; c) activation of the electrophile; and finally d) electrostatic stabilization of the transition state [1]. Additional mechanisms have been proposed for specific enzymes, including: proton relay systems [2], substrate-assisted catalysis [3] and strain of the β and γ phosphate groups [1]. Phosphoryl transfer enzymes are a broad class within which several mechanisms may have evolved. However, one is struck by the diversity in mechanistic proposals and the lack of consensus on key characteristics of active sites that might implicate common elements of mechanism that might bridge across diverse enzyme families.

**Figure 1:**
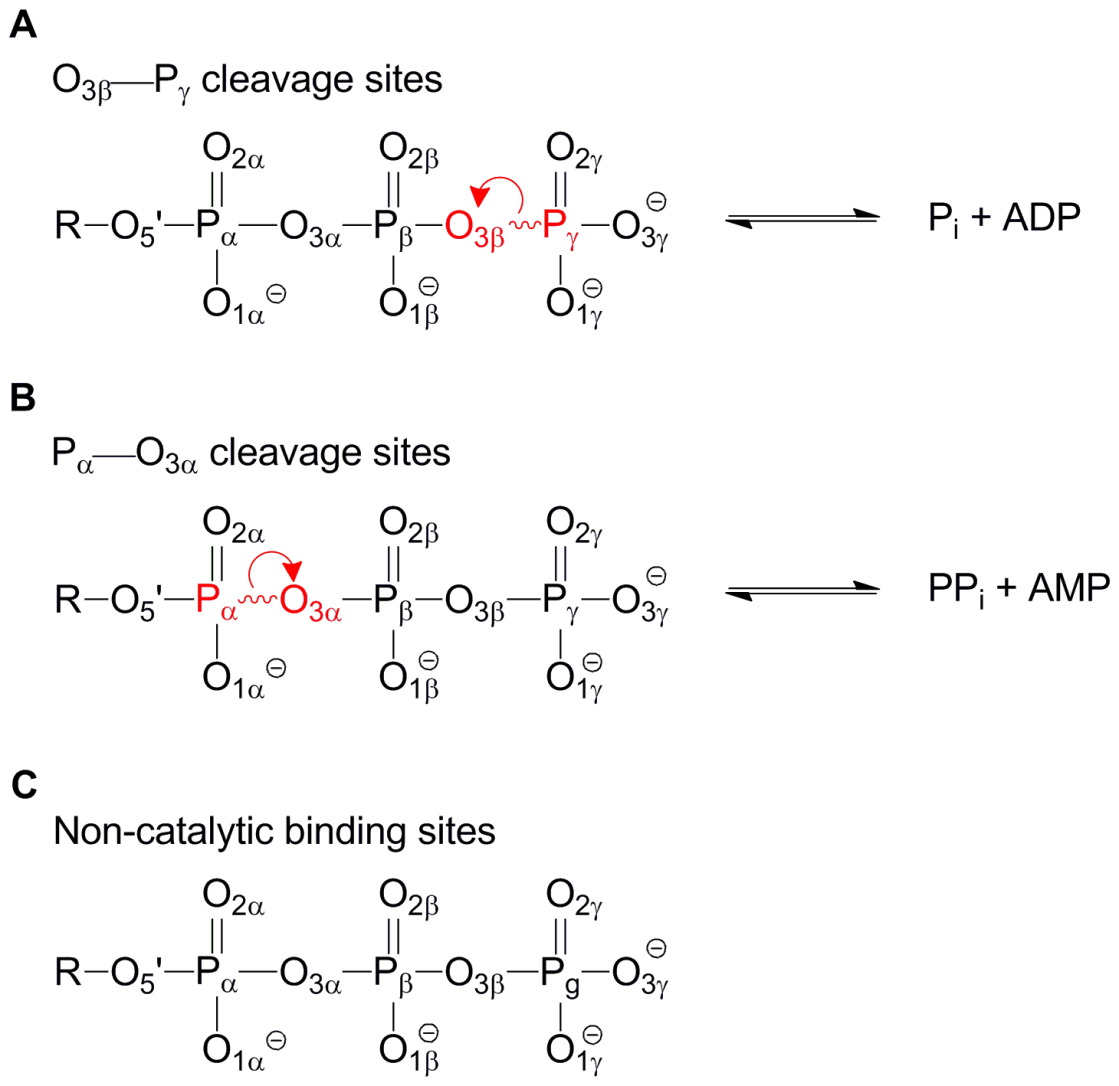
Enzyme structures can be categorized according to the fate of the bound nucleoside triphosphate (NTP). A) Phosphoryl transfer in which the O_3β_―P_γ_ bond is cleaved. B) Reactions in which the P_α_―O_3α_ bond is cleaved and C) Structures where the bound NTP does not undergo a chemical reaction. Red lettering indicates the atoms in the scissile bond and red arrows depict the transfer of electrons in going from reactants to products.

The Protein Data Bank (PDB) provides an opportunity for a top-down analysis of common active site configurations, including hydrogen bonding interactions with water. In this work, phosphoryl transferases are used to test the postulate that comparative analysis can complement traditional reductionist investigations of individual systems, providing additional enzymological insights. Three classes of structures are compared here: those that cleave an O_3β_—P_γ_ bond (Figure 1a); those that cleave a P_α_—O_3α_ bond (Figure 1b), and a “control” group of those that bind nucleotide without catalyzing phosphoryl transfer (Figure 1c). This work stems from the premise that important atomic interactions may have been hidden by the experimental error in the analysis of individual structures. We explore whether they can be revealed through statistical analysis of populations of structures of proteins sharing similar function, examining the spatial distributions of atoms at a local level. Our statistical analyses reveal that interactions with the bridging oxygen of a scissile phosphoanhydride bond, previously noted for select enzymes [2], [4], [5], [6], are present in diverse enzymes. Interactions with non-bridging β-phosphate oxygens are also widespread in active sites. The analysis is put to brief experimental test through kinetic analysis of an active site mutant affecting interactions with the bridging β-oxygen in the reaction of arginine kinase, an enzyme in which such interactions had not previously been implicated. Finally, quantum mechanical calculations reveal a possible stereochemical rationale for the observed interactions with both bridging and non-bridging β-oxygens, adding to a number of plausible proposals that seek to explain the bond selectivity and mechanism of phosphoryl transfer.

## Materials and Methods

### Database analysis

#### Structure sets

Coordinates for protein-nucleotide complexes (Table S1) were downloaded from the PDB, including those containing ATP, UTP, TTP, GTP, CTP or their analogs. Two sets of data were compiled, one containing structures up to 2.0 Å resolution, where solvent molecules were defined with confidence, and a larger group (inclusive of the first) up to 2.7 Å resolution from which solvent water was excluded, due to insufficient confidence in their positions at this resolution. Of the 1866 protein-NTP complexes to 2.7 Å, as of October 3^rd^, 2012, 305 were non-redundant and satisfied inclusion criteria (Table S2) that, for example, excluded structures in conformations known to be non-catalytic. Of the 305 vetted structures, 134 were at resolutions higher than 2.0 Å. Molprobity's Reduce [7] was used to flip asparagine, glutamine and histidine side chains for optimal hydrogen bonding.

**Figure 2:**
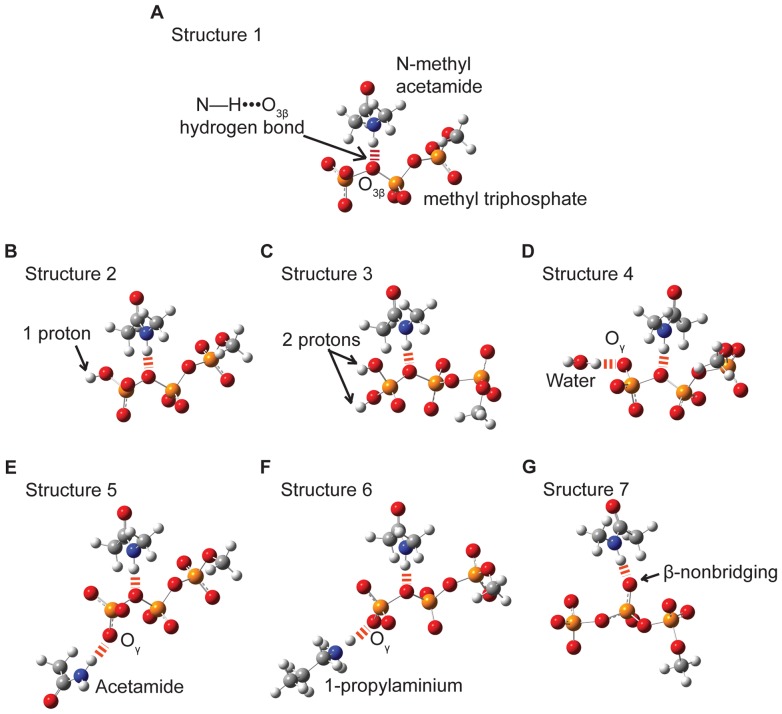
Models used to test the dependence of hyperconjugation and O_3β_―P_γ_ bond length on enzyme-ligand interactions. N-methylacetamide (A) was used to model a (neutral) protein backbone amide hydrogen bond to O_3β_, using methyl triphosphate to model an NTP nucleotide. Structures 2 (B) and 3 (C) were used to investigate the effects of protonation at the γ-oxygens. Additional active site hydrogen bonds, represented in Structures 4 through 6 (D–F), were used to assess the secondary effects of different types of O_γ_ hydrogen bonds. Acetamide (E) 1-propylaminium (F) were used to model asparagine and lysine side chains respectively. Structure 7 (G) was used to investigate the impact of hydrogen bonding at a nonbridging β-oxygen on hyperconjugation and O_3β_―P_γ_ bond length. Hydrogen bonds are shown with dashed red lines. White = hydrogen, gray = carbon, red = oxygen, blue = nitrogen, orange = phosphorus.

Structures were grouped by the site of bond cleavage in the ligand: 1) at the O_3β_—P_γ_ bond (Figure 1a); 2) at the P_α_—O_3α_ (Figure 1b); and 3) those in which the ligand binding site is non-catalytic (Figure 1c). The groups had 155, 100, and 50 PDB structures in the 2.7 Å set, respectively and 72, 45 and 17 structures in the 2.0 Å set. An underlying premise was that enzymes might share catalytic features regardless of base-type (GTP, ATP, *etc.*). The set of proteins with non-catalytic binding sites serves as a control to distinguish potentially catalytic interactions from those primarily involved in ligand-binding. Comparisons between the sets of enzymes with P_α_—O_3α_ and O_3β_—P_γ_ cleavage sites also help to distinguish binding and catalytic interactions, due to spatial separation of binding and catalytic sub-sites.

#### Identification of interactions

Coordinates were expanded according to the crystallographic symmetry and riding hydrogen atoms were added. These hydrogens had not been seen directly by x-ray crystallography, so their positions were inferred from the heavy atom coordinates using Reduce from the MolProbity program [7]. Assessed interactions included hydrogen bonds and coordination of metal ions, all analyzed using an in-house Python program.

Heavy atom distance and angle criteria were based on previous work [8]: O•••D distance ≤3.5 Å, P—O•••D angle ≥90°, where D is the hydrogen bond donor, P is the ligand phosphorus atom, and O is the oxygen atom of the ligand. An additional criterion was added: O•••D—R angle ≥90° where R is the antecedent atom of a hydrogen bond donor. These criteria depend only on the heavy atoms directly observable and can therefore be applied directly to medium resolution crystal structures. Assessments were also made using additional criteria possible once riding hydrogens were added: O•••H≤2.7 Å, P—O•••H≥80° and O•••H—D≥80°. Inspection revealed that a small number of atom pairs satisfied the criteria without interacting directly, because they were brought into proximity by coordinating with a common metal ion. These were excluded using criteria shown in Figure S1. Metal interactions were subject only to a distance cut-off of 2.8 Å derived from analysis of metal coordination in the Cambridge Structural Database.

#### Classification and comparison of interactions

Ligand oxygens were organized into six groups: Non-bridging γ-, β- and α-oxygens, and bridging β-, α- and O5′-oxygens. Distributions of enzyme-ligand interactions for each of the groups were compared using pair-wise two-tailed t-tests in RStudio using pooled standard deviations. When comparing the numbers of interactions with different atoms for the different structure sets, the Benjamini & Hochberg correction for multiple comparisons was applied. For each comparison, the null hypothesis was tested that the two sample means could be drawn from the same population, at a significance level of α = 0.05. Results are fully tabulated in the supplemental material (Table S3 and Table S4).

### Quantum mechanical analysis of interactions

The Gaussian 09 program [9] was used for all electronic structures calculated with the B3LYP/6-311++G(d,p) level of theory [10]. Bulk solvent was modeled implicitly with the polarizable continuum model (PCM) using a solvent dielectric of 78.36 for water [11]. This level of theory had previously been shown to be appropriate for capturing solvation effects [12]. Natural bond orbital (NBO) analysis [13] was performed with the GenNBO5.0 program [14] using the B3LYP/6-311++G(d,p)-optimized structures and HF/cc-PVTZ model chemistry [15]. The NBO program facilitates a Lewis-like description of electron density by transforming nonorthogonal atomic orbitals from a HF wave function into natural bond orbitals. Second order perturbation E(2) energy, given by Equation 1, estimates the magnitude of electron delocalization and hyperconjugation.
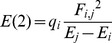
(1)q_i_ is the donor orbital occupancy, and E_j_ – E_i_ is the energy gap between donor orbital i and acceptor orbital, j. F_i,j_ is the Fock matrix element which describes donor and acceptor orbital overlap. In this work, the donor will be a lone pair orbital of the γ-phosphate oxygens and the acceptor orbital is σ*(O_3β_—P_γ_), the antibonding orbital of the O_3β_—P_γ_ bond in methyl triphosphate. Methyl triphosphate has been used previously, to model interactions in ATP (Figure 2) [16].

The influence of a N—H•••O_3β_ hydrogen bond on the O_3β_—P_γ_ bond length and hyperconjugation was evaluated with Structures 1 through 6 (Figure 2a–f). (Similar results were obtained with the addition of Mg^++^ coordinated to β- and γ-oxygens.) Structure 7 was used to evaluate the effects of a hydrogen bond to a nonbridging β-oxygen (Figure 2g). N-methylacetamide was used to model a peptide backbone (neutral) hydrogen bond donor. The N—H•••O_β_ hydrogen bond was weakened by increasing its length from 2.5 Å to 3.5 Å in 0.1 Å increments for Structures 1 through 3 and in 0.2 Å increments for Structures 4 through 7. These changes were made by translating the N-methylacetamide, fixing the O_3β_ and N atoms at a specific hydrogen bond distance, then optimizing other aspects of the structure with the N—H•••O_β_ angle fixed at 170°. Structures 2 through 6 were used to probe the electronic effects of either protontion or hydrogen bonding at the γ-oxygens of methyl triphosphate (Figure 2b–f). For protonated structures the O_3β_—P_γ_—O_γ_—H dihedral angle was fixed at 180°. Acetamide was used as a model for an asparagine side chain in Structure 5, and 1-propylaminium was used as a model for a lysine side chain in Structure 6. All hydrogen bonds to γ-oxygens, were fixed at a D•••O_γ_ distance of 2.8 Å, where D is the hydrogen bond donor heavy atom. The D•••O_γ_—P_γ_—O_3β_ and the (D)H•••O_γ_—P_γ_—O_3β_ dihedral angles were each fixed at 180°. Changes in O_3β_—P_γ_ bond lengths and in E(2) energies were calculated relative to each molecule without a hydrogen bond between N-methylacetamide and O_β_.

### Mutagenesis and Kinetics

Mutants of *Limulus polyphemus* arginine kinase (AK) were made using the QuikChange mutagenesis kit (Stratagene, Inc.). Expression, purification and kinetic analysis of mutants R280L and R280K followed previously published methods [17], though only R280K had sufficient activity for full analysis. Initial velocities were measured using a 6×6 grid of substrate concentrations: ATP at 0.2, 0.4, 0.8, 1.2, 1.6, and 2.0 mM, and arginine at 0.2, 0.4, 0.8, 1.6, 3.2, and 7.0 mM. Duplicate measurements were made with separate protein preparations. Steady state parameters K_ia_, K_m_ and V_max_ for a random order sequential bimolecular-bimolecular (bi-bi) reaction mechanism were fitted to the data by nonlinear least squares using SigmaPlot.

## Results & Discussion

### Database analysis of enzyme-nucleotide interactions

Nucleotide interactions included hydrogen bonds with the enzyme, water or other substrates, as well as interactions with metal ions. The numbers of interactions were compared between phosphoryl transfer enzymes (Figure 1a) and two comparison groups (Figure 1b, c) to distinguish potentially catalytic from non-catalytic associations. T-tests reveal two overall trends (Table S3 and Table S4), that interactions with both non-bridging β-oxygens and with the bridging oxygen of a scissile phosphoanhydride bond are more prevalent in phosphoanhydride bond-cleaving sites than in non-catalytic nucleotide-binding sites.

**Figure 3:**
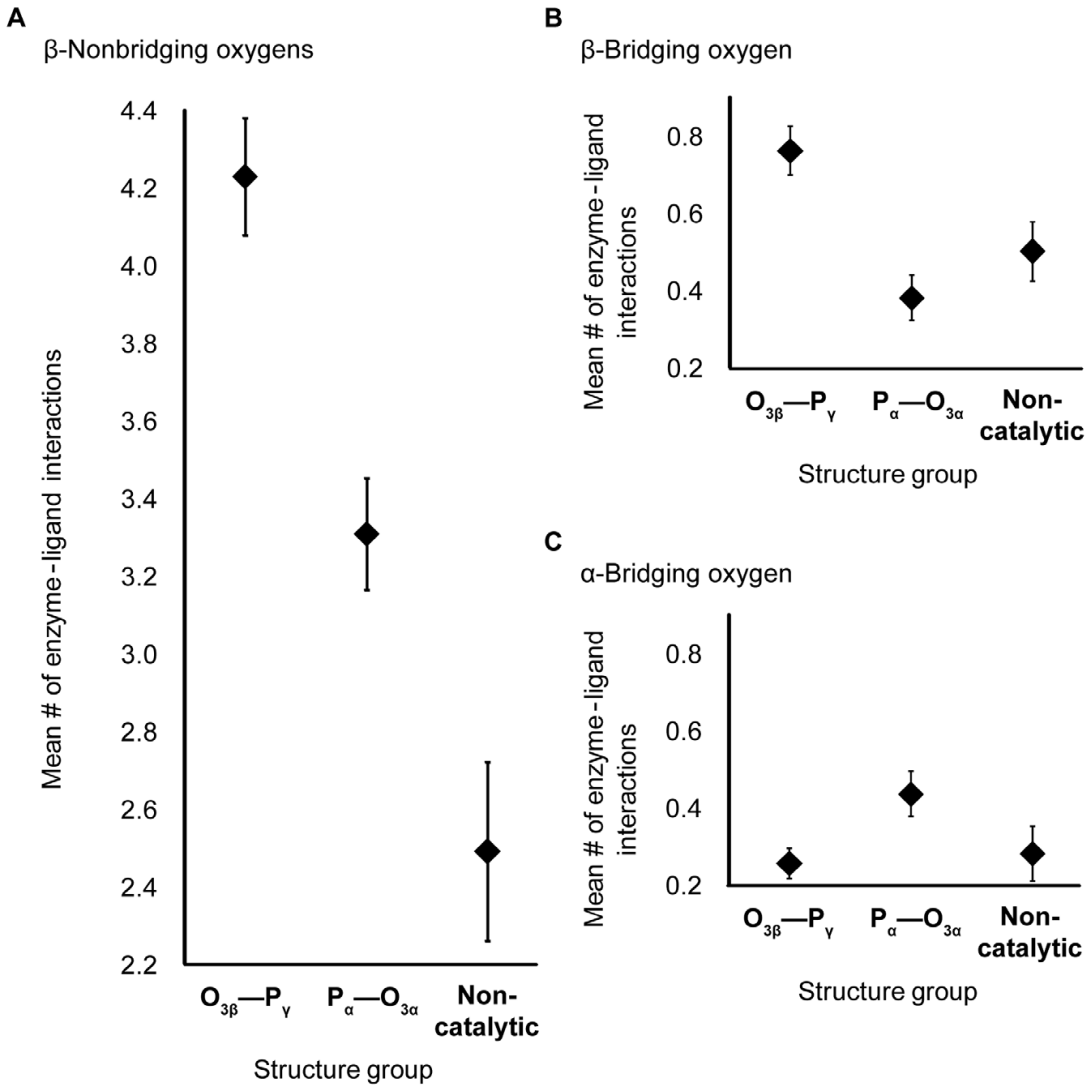
Mean number of enzyme-ligand interactions. Interactions are shown with nonbridging β- (A), bridging β- (B) and bridging α-oxygens (C) in the O_3β_―P_γ_ cleaving-, P_α_―O_3α_ cleaving- and non-catalytic-NTP-binding sites. The resolution cutoff of structures used is 2.7 Å, and water is not included. Bars show standard errors.

For non-bridging β-oxygens, the mean numbers of interactions in both O_3β_—P_γ_ –cleaving sites (μ = 4.2, σ = 0.2) and P_α_—O_3α_–cleaving sites (μ = 3.3, σ = 0.1) are significantly greater than in non-catalytic sites (μ = 2.5, σ = 0.2; p<0.05; 2.7 Å; Figure 3a, Table S3a). The database analysis extends the finding in Ras, by Fourier Transform Infrared (FTIR) spectroscopy, of stronger interactions with β-oxygens than α- or γ-oxgyens in [18], indicating that strong O_β_ interactions are widespread among enzymes. Enzymes that cleave the O_3β_—P_γ_ bond favor neutral H-bond partners with the non-bridging β-oxygens, whereas positively charged hydrogen bond donors are common in non-catalytic NTP binding sites. Backbone amide O_β_ interactions have been implicated with Walker A motif P-loops [1], [19], so the pre-eminence of neutral hydrogen bonding in the database indicates that analogous interactions are favored in a more general array of phosphoryl transfer enzymes.

Although not every protein interacts with the bridging O_3β_, interactions are about twice as common in O_3β_—P_γ_ cleaving sites (mean number of 0.8, σ = 0.1) as in either P_α_—O_3α_ cleaving sites (μ = 0.4, σ = 0.1) or non-catalytic NTP-binding sites (μ = 0.5, σ = 0.1; p<0.05; 2.7 Å; Figure 3b, Table S3a). (The statistics quoted are for the larger data set, but similar results are also obtained with the smaller 2.0 Å set that included solvent waters, Table S3b and Table S4b). Hydrogen bonds with the O_3β_ most commonly involve neutral donors, particularly from backbone nitrogens (Table S5). Other donors include water and positively charged amino acids, especially arginine.

Interactions with bridging O_3β_ have previously been cited for G proteins and several kinases, including 6-phosphofructo-2-kinase/fructose-2,6-*bis*phosphatase and pyruvate kinase [2], [5], [20]. Thus, the structural survey captures results expected from detailed prior analyses and shows that analogous interactions actually occur in a diverse array of phosphoryl transfer enzymes including the MutL DNA mismatch repair protein [21], the DEAD-box RNA helicase [22], and Phosphoenolpyruvate carboxykinase [23]. The donor is often, but need not be, within some type of conserved motif, for example at position 4 of the Walker A motif (p-loop; Table S5). O_3β_ interactions have previously been rationalized in terms of stabilizing development of negative charge on the leaving group in proposed dissociative transfer mechanisms [2], [5], [20], though Warshel has noted that associative mechanisms would likely also benefit from electrostatic stabilization of increased electron density on the O_3β_ [24], [25]. Our survey demonstrates O_3β_ interactions occur in diverse enzyme families, such as the phosphagen kinases, N-Acetyl-L-Glutamate kinase and Adenosine kinase, in which the mechanisms have been reported to be significantly associative [26], [27], [28], [29]. Thus, the interaction may transcend the associative/dissociative mechanistic categorization. The expanded set of enzymes exhibiting O_3β_ interactions represent a wide array of protein folds and differ in other aspects of active site configuration. Indeed, the proposed primary catalytic mechanisms of these enzymes are quite diverse, so it was not obvious that such a broad array of enzymes would share analogous interactions. The much greater incidence of O_3β_ interactions among enzymes cleaving at O_3β_—P_γ_ over P_α_—O_3α_, and over proteins with non-catalytic nucleotide binding sites, suggests that most of these interactions have evolved to have a role in the chemistry and not just binding.

Interactions with O_3α_ in enzymes that cleave the P_α_—O_3α_ bond are more frequent than in the comparison groups, but more marginally than the O_3β_ interactions. Only the difference between P_α_—O_3α_ cleaving sites (μ = 0.4, σ = 0.1) and O_3β_—P_γ_ cleaving sites (μ = 0.3, σ<0.1) reaches statistical significance (p<0.05, Table S3a).

**Figure 4:**
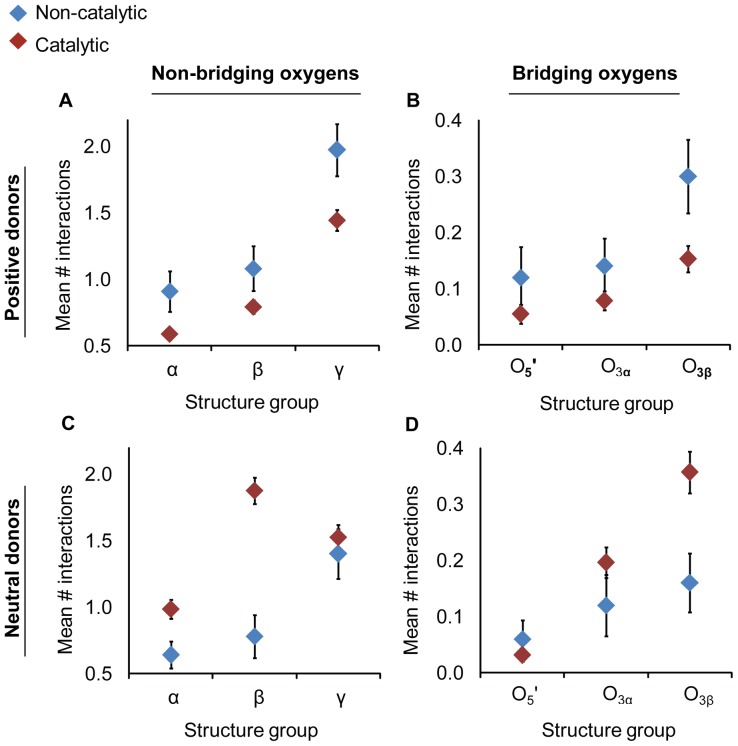
Non-catalytic (blue) active sites have a preference positively charged hydrogen bond donors (a–b) whereas in catalytic (red) active sites neutral interactions are favored (c–d). Mean numbers of hydrogen bonds were measured for non-bridging (a & c) and bridging (b & d) oxygens. The catalytic group is composed of both O_3β_—P_γ_ and P_α_—O_3α_ structure sets. Positive donors include Lys, Arg, and His side chains and neutral donors include Asn, Gln, Trp, Ser Thr,Tyr, Cys side chains, the nucleotide O2′ and O3′ oxygens and all backbone nitrogens (except Pro).

A preference was found for positively charged hydrogen bond donors in non-catalytic binding sites relative to the catalytic sites (O_3β_—P_γ_ and the P_α_—O_3α_ combined group; Figure 4a, b). Differences manifested particularly at the non-bridging γ-oxygens (catalytic: μ = 2.0, σ = 0.2; non-catalytic: μ = 1.4, σ = 0.1; p<0.05; 2.7 Å; Table S6)). The number of neutral hydrogen bond donors in catalytic sites exceeded the average in non-catalytic sites (Figure 4c, d) particularly for the non-bridging (catalytic: μ = 0.8, σ = 0.2; non-catalytic: μ = 1.9, σ = 0.1) and bridging β- oxygens (catalytic: 0.2, σ = 0.1; non-catalytic: μ = 0.4, σ<0.1; p<0.05; 2.7 Å; Table S6). There are several possible explanations for the observed differences in disposition of charged interactions, including the possibility that non-catalytic binding sites have evolved without pressure for efficient dissociation of reaction products.

### Quantum mechanical modeling of nucleotide interactions

Several mechanistic rationales have been offered for the O_3β_ interactions in G proteins and related enzymes [2], [4], [5], [6], [20], [25], [28], [30], [31], [32]. This section examines if such interactions could (also) affect nucleotide stereoelectronics in light of its recently appreciated importance in the modulation of phosphoanhydride bond energies [12], [16]. The total of hyperconjugative effects has been estimated at ∼300 kJ/mol, with hydrogen bonds to γ-oxygens each lessening this by ∼20 kJ/mol [16], so modulation of active site interactions could plausibly impact reactions with activation barriers like the 34 kJ/mol of arginine kinase [17]. Our approach is Natural Bond Order analysis [13] which can inform us of the ground state disposition, but is not applicable to the transition structure, so the approach allows only limited inferences about rate enhancement.

**Figure 5:**
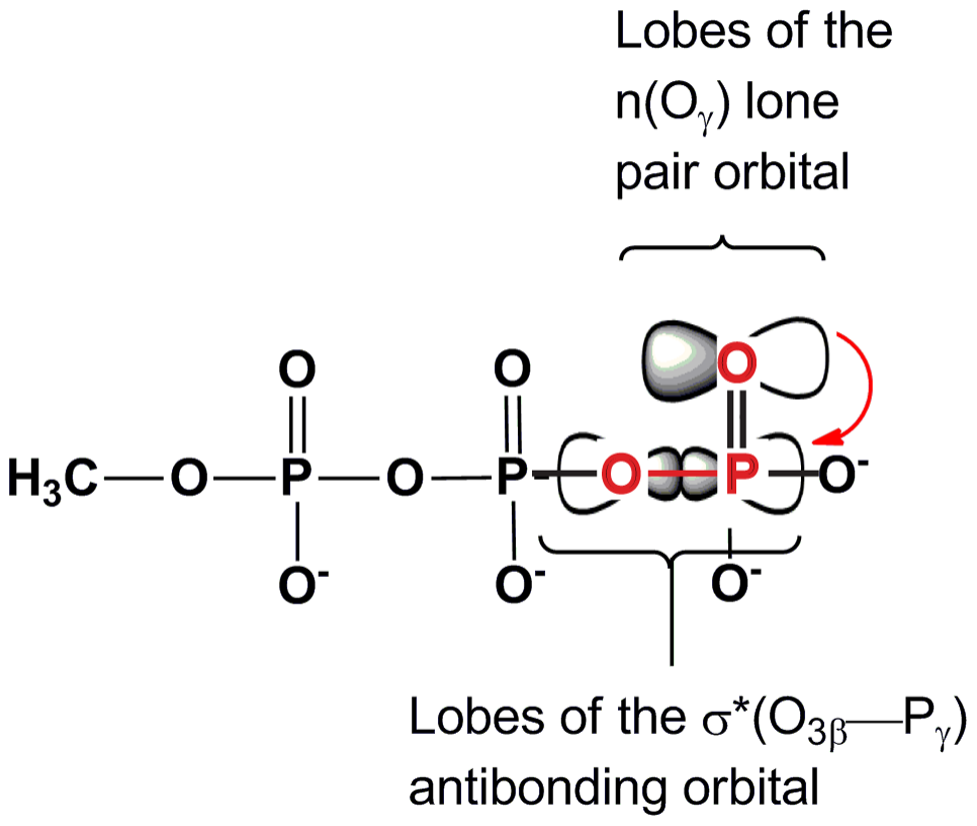
Hyperconjugation in methyl triphosphate, a model for ATP. Electron density is transferred from a lone pair orbital on one of the γ-oxygens (O_γ_), into the antibonding orbital (σ*) of the O_3β_—P_γ_ bond. Red lettering indicates the atoms involved in the hyperconjugative interaction and the red arrow represents electron density transfer.

For G proteins, the O_3β_ interactions have been rationalized through electrostatics [5]. Hydrogen bonding or cation interactions would favor charge accumulation on O_3β_ in the progression towards a dissociative transition state, or it could favor the transient accumulation of electron density in an associative mechanism [24], [25]. It has also been noted that favoring charge accumulation on O_3β_ would also tend to increase the susceptibility of P_γ_ (and P_β_) to nucleophilic attack [28]. A dissociative mechanism has been cited as consistent with FTIR experiments on Ras-GAP [33] that indicated reduction of β- and increase in γ-phosphoryl group bond orders. It is thought that electrostatic stabilization of negative charge at the β-oxygens draws negative charge (by induction) out of the O_3β_—P_γ_ bond onto the O_3β_ oxygen, which is then partially transferred to the non-bridging β-oxygens through resonance [33], [34], [35]. A small molecule quantum study of a series of organic phosphates indicated, however, that hyperconjugation was likely to be a greater factor than induction in scissile bond lengths [12]. Hyperconjugation is a stereoelectronic effect, where electron density is transferred from an electron rich donor orbital to the antibonding orbital of a neighboring bond [36]. An example is depicted in Figure 5, where electron density is transferred from a γ-oxygen lone pair orbital in methyl triphosphate into the antibonding orbital of the O_3β_—P_γ_ bond. Rationalization of the mechanistic impact of O_3β_ interactions has already been controversial [5], [24], [25], [28]. The goal here was not to arbiter this discussion, but to examine the plausibility of O_3β_ interactions having impact mediated through hyperconjugation, possibly in addition to a mixture of other effects.

Past quantum mechanical modeling of methyl triphosphate [12], [16] has shown that enhanced hyperconjugation renders γ-oxygens more positive and the O_3β_ more negative as electron density is transferred from the γ-phosphoryl group to the σ* O_3β_—P_γ_ antibonding orbital, lengthening the scissile bond. Qualitatively, the changes in charge and bond length are the same as those proposed for electrostatic O_3β_ interactions [5] and observed through FTIR on RAS-Gap [33]. Thus the task is not to rule out the possibility of electrostatic contributions to the mechanism but rather to determine whether hyperconjugation could be a major contributor as well. Whether interactions with the β-oxygens can enhance hyperconjugation in a nucleotide, is tested through quantum mechanical calculations on a methyl triphosphate model system with a hydrogen bond to one of its β-oxygens from N-methylacetamide (Figure 2). The dependence of hyperconjugation upon the N—H•••O_3β_ hydrogen bond is investigated by calculation of the O_3β_—P_γ_ bond length as the length of the N—H•••O_3β_ hydrogen bond is perturbed.

**Figure 6:**
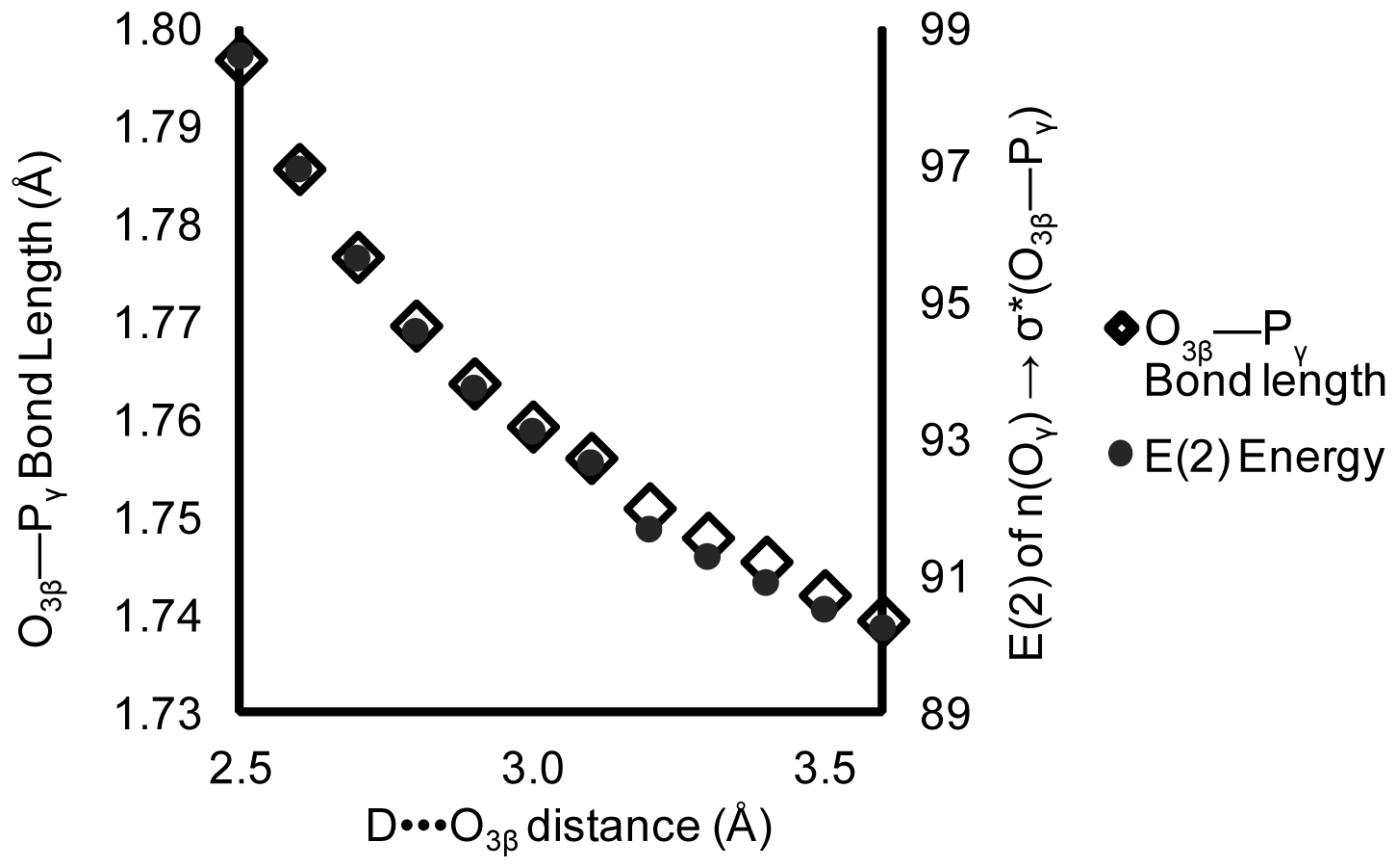
The O_3β_—P_γ_ bond length and hyperconjugation increase with decreasing D•••O_3β_ hydrogen bond length in Structure1. Calculated E(2) energy is of the n(O_γ_)→σ*(O_3β_—P_γ_) hyperconjugative interaction. D is the N—H hydrogen bond donor in N-methylacetamide.

Density functional calculations on Structure 1 (Figure 2a) show that a single hydrogen bond, between N-methylacetamide and the O_3β_ oxygen in methyl triphosphate, induces an increase in hyperconjugation and a ∼0.05 Å elongation of the O_3β_—P_γ_ bond (Figure 6). This affirms the hypothesis that the O_3β_—P_γ_ bond length can, at least in principle, be weakened by O_3β_ hydrogen bonding acting through hyperconjugation.

**Figure 7:**
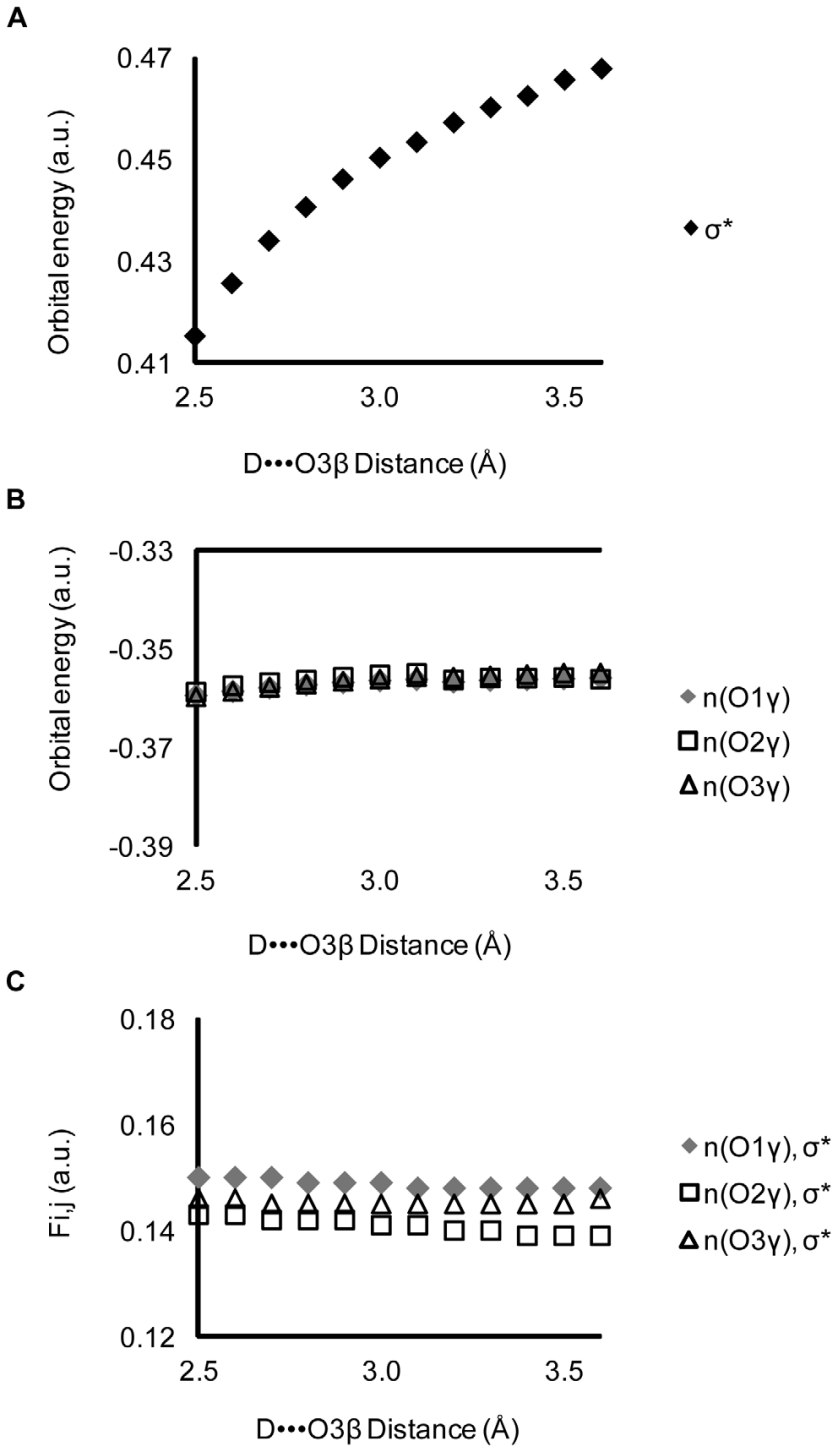
Effects of a hydrogen bond at O_3β_ on orbital and interaction energies in Structure1. Shortening the hydrogen bond between N-methylacetamide and methyl triphosphate: A) decreases the orbital energy of the σ*(O_3β_—P_γ_) anti-bonding orbital; while (B) leaving unchanged both the n(O_γ_) donor orbital energies and (C) F_i,j_, a measure of the overlap between the n(O_γ_) lone pair orbitals and σ*(O_3β_—P_γ_). D denotes hydrogen bond donor. σ* denotes σ*(O_3β_—P_γ_).

Two factors determine the magnitude of hyperconjugation; the degree of donor and acceptor orbital overlap, and the orbital energy gap. The orbital overlap (F_ij_ in Eq. 1) and the orbital energies of n(O_γ_) and σ*(O_3β_—P_γ_) were calculated for Structure 1 (Figure 2a), as a function of hydrogen bond length (Figure 7). There is a strong dependence of the energy of the σ*(O_3β_—P_γ_) antibonding orbital upon strength of the hydrogen bond (Figure 7a). However, neither the energy of the non-bridging γ-oxygen lone pair orbitals (Figure 7b) nor the orbital overlap (Figure 7c) are affected. As the hydrogen bond is shortened (made stronger), the orbital energy gap decreases, increasing hyperconjugation and elongating the O_3β_—P_γ_ bond.

**Figure 8:**
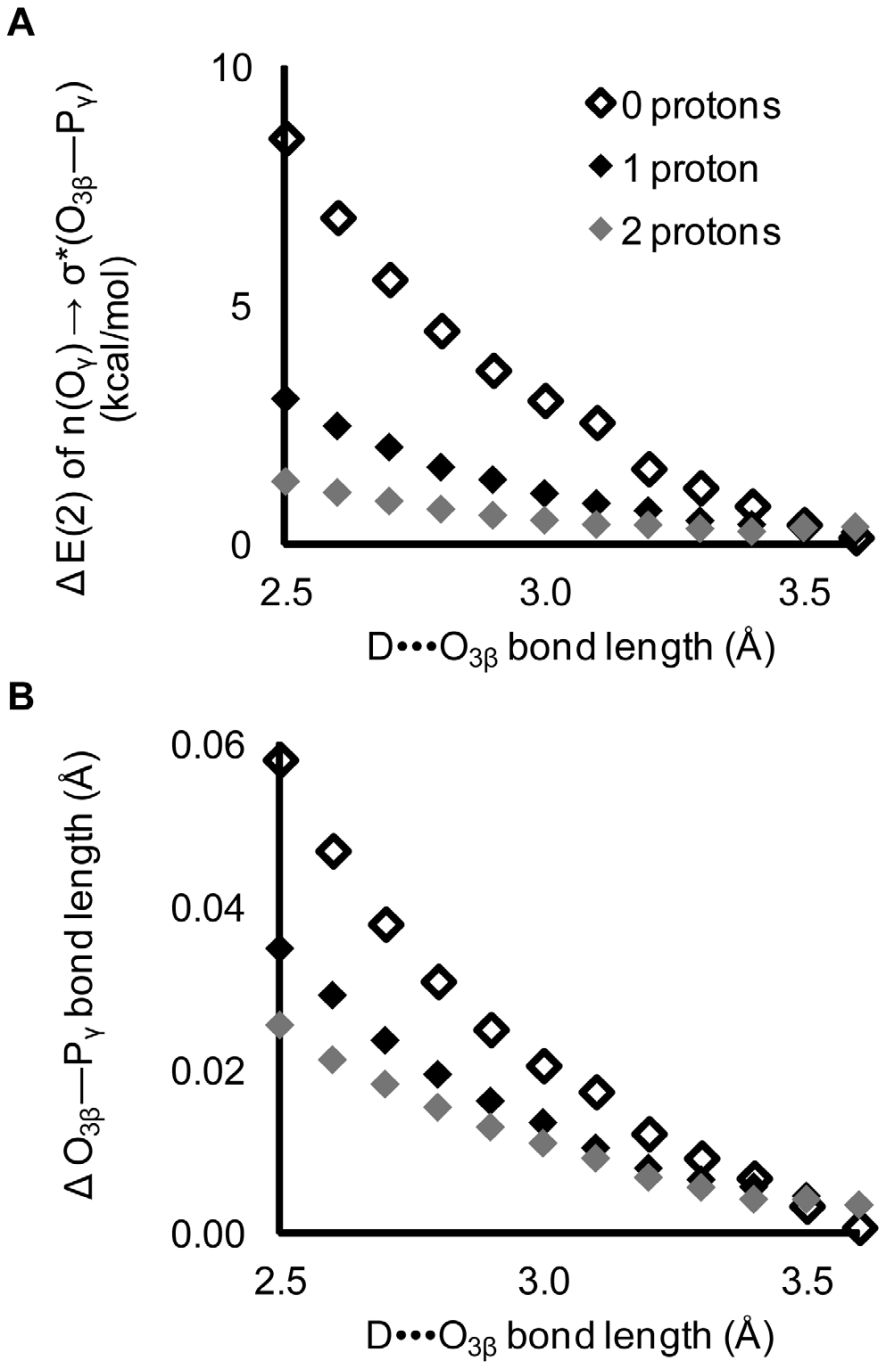
Impact of protonation of the γ-oxygens. Protonation at the γ-oxygens decreases the effects of the O_3β_ hydrogen bond on both A) the magnitude of hyperconjugation and B) the O_3β_—P_γ_ bond length. Changes are calculated relative to corresponding structures without a D•••O_3β_ hydrogen bond. The ability of interactions with the O_3β_ to increase the scissile bond depends substantially on the presence of strong intrinsic hyperconjugation in the ligand.

The relationship between O_3β_—P_γ_ bond elongation and hyperconjugation was confirmed by protonating the γ-oxygens. Protonation of the γ-oxygens decreases the hyperconjugative interaction: n(O_γ_)→σ*(O_3β_—P_γ_) [12], [16]. We now find that protonation also decreases the dependence of the O_3β_—P_γ_ bond length on the strength of the D•••O_3β_ hydrogen bond (Figure 8), consistent with the impact of an O_3β_ hydrogen bond upon O_3β_—P_γ_ elongation being significantly mediated by hyperconjugation.

Hyperconjugation is also subject to other “secondary” perturbations that might be of greater biochemical relevance; hydrogen bonds added at the γ-oxygens interfere with hyperconjugation [16]. The primary dependence of O_3β_—P_γ_ elongation on the strength of the O_3β_ hydrogen bond was then recalculated, after the addition of (secondary) hydrogen bonds at γ. Several γ-oxygen hydrogen bond donors were considered: water, acetamide (mimicking an asparagine) and 1-propylaminium (mimicking a lysine; Figure 2). Hydrogen bonds to γ-oxygens were fixed at 2.8 Å, while the hydrogen bond between N-methylacetamide and the O_3β_ oxygen was varied from 2.5 Å to 3.1 Å in 0.2 Å increments.

**Figure 9:**
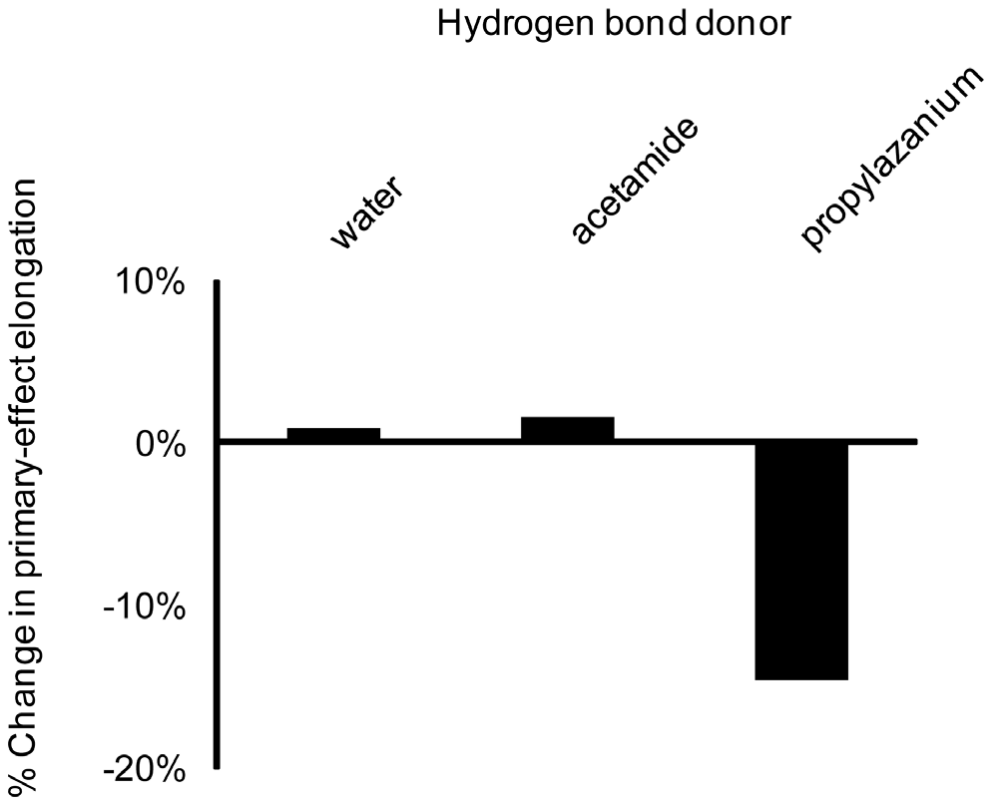
Impact of (secondary) interactions with γ-oxygens on O_3β_—P_γ_ bond elongation induced by (primary) O_3β_ interactions. Secondary interactions with γ-oxygens have modest impact (much smaller than the direct effects characterized by Summerton et al. [16]) and are substantial only for charged donors.

Hydrogen bonds from neutral donors (water or acetamide) to a γ-oxygen have little effect (Figure 9). Only the salt bridge bond from a positively charged 1-propylaminium donor modulates the primary interactions. The effect is modest, reducing bond elongation by about 15%, but it is possible that multiple O_γ_ salt bridges could have an additive effect.

*Quantum mechanical analysis of interactions with non-bridging oxygens* – Of interest is whether hyperconjugation can explain the higher frequencies of interactions with non-bridging β-oxygens in O_3β_—P_γ_ cleaving active sites, relative to non-catalytic binding sites (Figure 3). Similar to our study of O_3β_ interactions, QM calculations were performed for N-methylacetamide hydrogen-bonded to a non-bridging β-oxygen in Structure 7 (Figure 2g). Changes in the n(O_γ_)→σ*(O_3β_—P_γ_) hyperconjugative interaction were calculated as the hydrogen bond length was varied from 2.5 Å to 3.5 Å in 0.2 Å increments.

**Figure 10:**
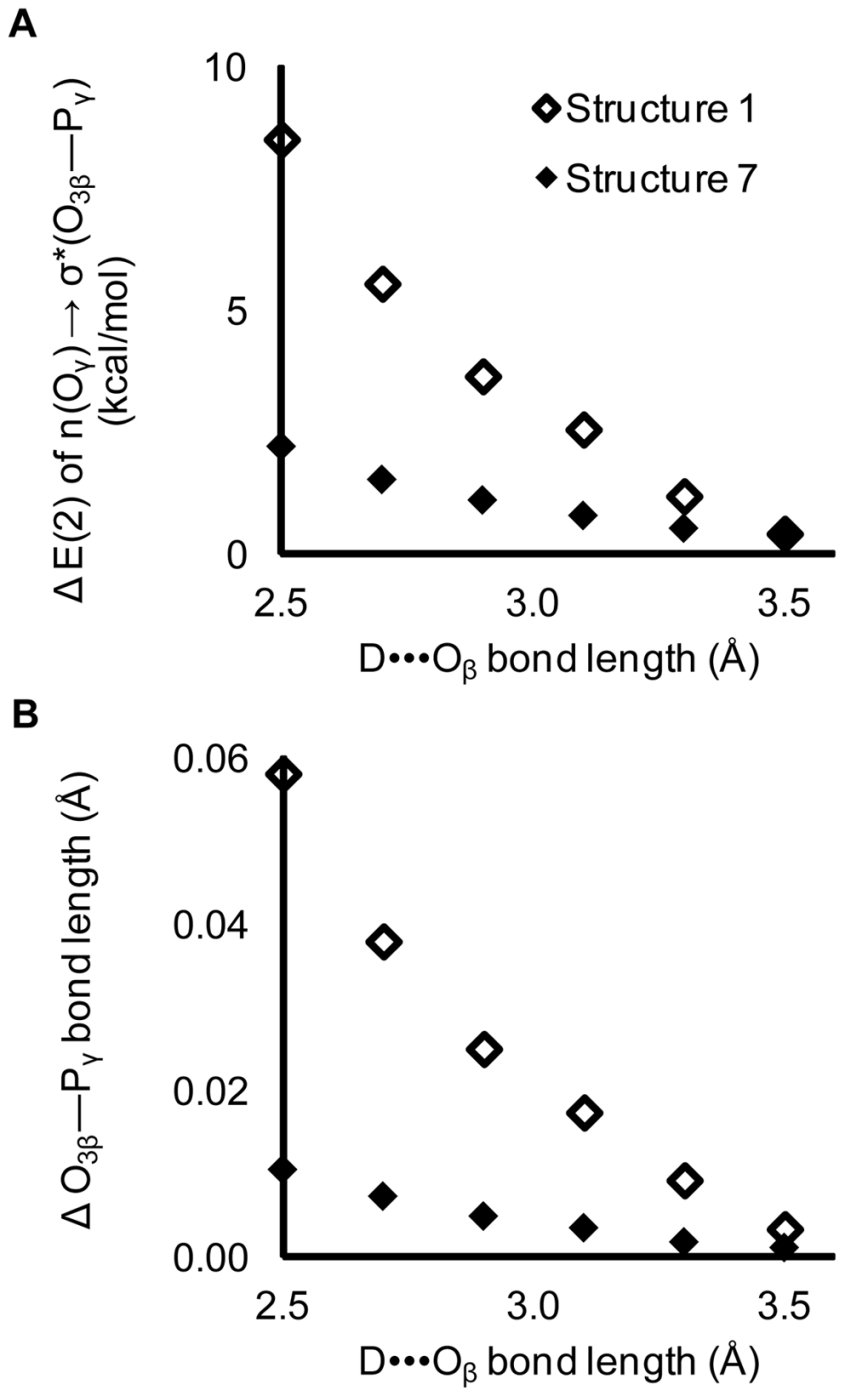
Impact of a hydrogen bond with a non-bridging β-oxygen. An interaction between N-methylacetamide and a non-bridging β-oxygen of methyl triphosphate decreases (A) the hyperconjugation and (B) scissile bond length. Interactions with the non-bridging β-oxygen (filled diamonds, Structure 7) are compared to those for O_3β_ (open diamonds, Structure 1). Changes are measured relative to methyl triphosphate absent a hydrogen bond.

The impact of a single hydrogen bond with a non-bridging β-oxygen is smaller than that of an O_3β_ hydrogen bond (Figure 10). However, the database analysis revealed an average of four enzyme interactions summed across the two non-bridging β-oxygens (Figure 3a), nearly saturating the lone pairs. Multiple hydrogen bonds to a single oxygen in methyl triphosphate had an additive effect on hyperconjugation [16]. Thus the aggregate effect of interactions with non-bridging β-oxygens could be commensurate with those of the O_3β_.

In summary, hyperconjugation weakens the scissile O_3β_—P_γ_ bond. Hydrogen bonds to the phosphate oxygens can either amplify or reduce this effect, according to proximity with the scissile bond. The greatest weakening of the scissile bond comes from interactions with O_3β_, proximal to the scissile bond, with lesser weakening from interactions at non-bridging O_β_ that are two bonds removed. By contrast, hydrogen bonds to γ-oxygens, vicinal to the scissile bond, interfere with the hyperconjugation, strengthening the scissile bond [16]. Changes in hydrogen bonding are inherent to the act of substrate binding, but of interest is whether particular types of interactions have been selected in enzyme evolution. Here we test the hypothesis that enzymes enhance hyperconjugation by maximizing binding to β- and minimizing binding to γ-oxygens.

**Figure 11:**
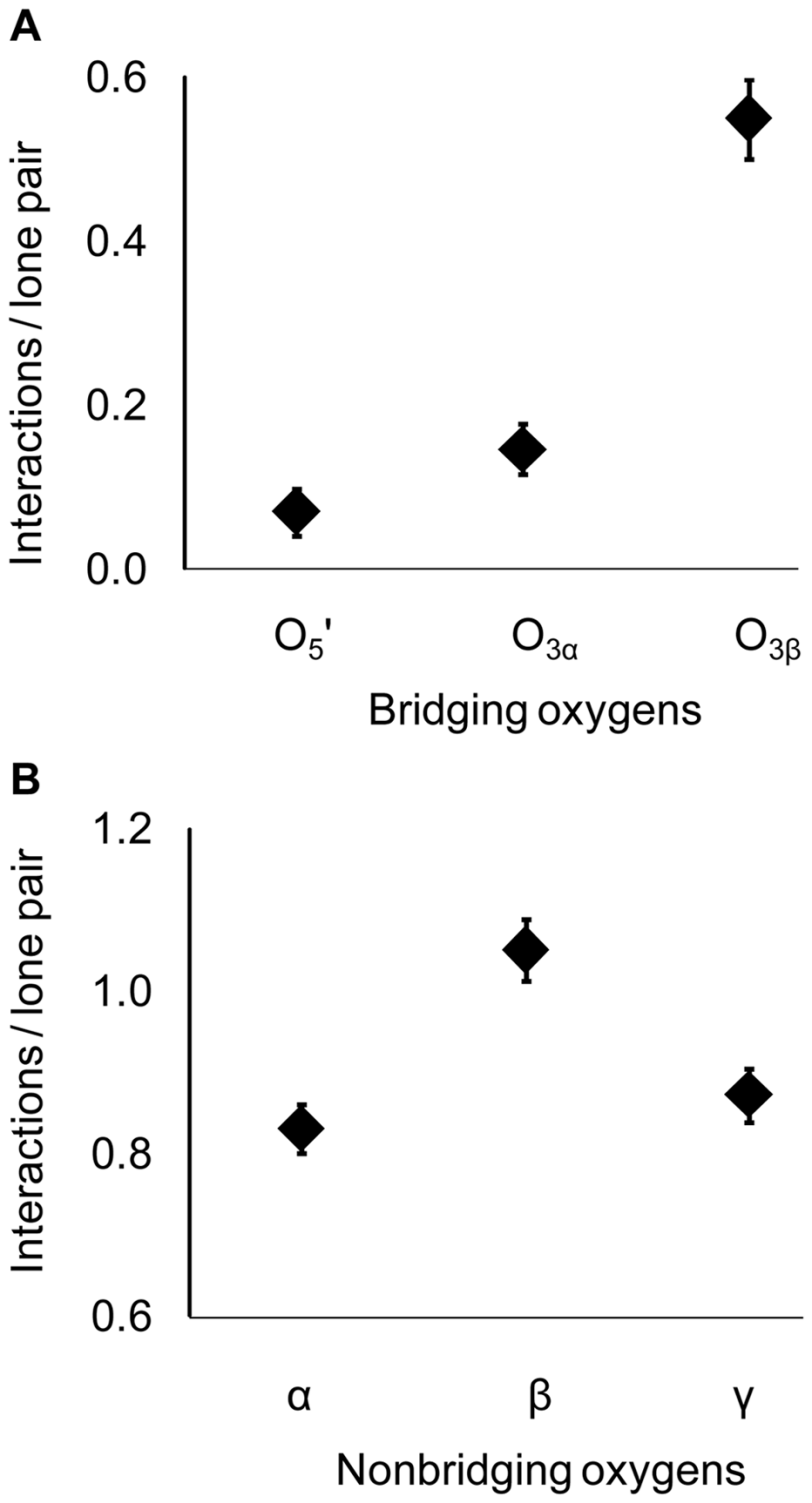
Saturation of NTP oxygen lone pairs by enzyme or solvent interactions in O_3β_—P_γ_-cleaving active sites. Bars denote standard errors.

Noting that the desolvation of the non-bridging β- and γ-oxygens on substrate binding would have opposite effects upon O_3β_―P_γ_ bond elongation, the 2 Å structure database was queried for the fractional saturation of lone pairs with enzyme or solvent interactions, comparing the total five lone pairs of non-bridging β-oxygens to the 8 of non-bridging γ-oxygens. As predicted, the non-bridging β-oxygens retain higher numbers of interactions than either the non-bridging α- or γ-oxygens (Figure 11, Table S7), and there are more numerous interactions with the bridging O_3β_ than either the O_3α_ or O_5′_. The favoring of interactions with the β-phosphoryl group that we see in the database analysis is consistent not only with the QM-based prediction, but with the FTIR studies of Ras that showed stronger binding of the β-phosphoryl group [37].

Consistency of the observed disposition of active site interactions makes a circumstantial case for the importance to enzymes of hyperconjugation-mediated weakening of the O_3β_—P_γ_ bond. The ground state QM calculations show clearly that bond selectivity is impacted, so enzymes could be taking advantage of hyperconjugation in specifying which of the phosphoanhydride bonds is to be cleaved. Understanding whether modulation of hyperconjugation has a role in rate enhancement is a more challenging question about which we cannot be as definitive. Bypassing the question of rate enhancement, even a role in bond selectivity alone could be of sufficient selective advantage to explain the prevalence of hyperconjugation-mediating interactions in the structural database.

Ground state quantum mechanical modeling provides, through hyperconjugation, a unifying rationalization for interactions with the phosphate oxygens. The calculated changes in bond order and partial charge are qualitatively consistent with those inferred from kinetic isotope and FTIR studies [33], [34], and are consistent with the dispositions of β- and γ-oxygen interactions seen in the database analysis. Hydrogen bonding at the bridging O_3β_ and non-bridging β-oxygens closes the energy orbital gap, increasing the transfer of electron density into the σ* antibonding orbital and lengthening the O_3β_―P_γ_ bond, while interactions at γ-oxygens have the opposite effect. Thus, differences in the interactions with β- and γ-oxygens can modulate the strength of the scissile bond in a mechanism mediated through hyperconjugation.

To affect rate, the activation barrier must be lowered by some combination of stabilizing the transition state (TS) or destabilizing the ground state. The (spontaneous) existence of hyperconjugation in isolated nucleotides means that it can be stabilizing the ground state. However, inter-molecular interactions that increase hyperconjugation beyond its natural equilibrium level would destabilize the substrate. Yang and Cui examined whether the anomeric effect in myosin destabilized the ground state [38]. A modest increase in ATP calculated potential energy, as the P_γ_—O_3β_ bond was lengthened, was deemed insufficient for substantial rate enhancement. However, the argument implicitly depends on a reciprocal relationship between bond length and hyperconjugation, such that distortions of the bond induce a change in hyperconjugation to the corresponding level of the enzyme-bound substrate, a questionable assumption. A definitive assessment of the influence of hyperconjugation on transition state stabilization, has been stymied by inapplicability of NBO calculations to the TS, and by flat energy surfaces (calculated for myosin and RasGAP reactions) from which it is difficult to distinguish different possible mechanisms and estimate entropic terms [25], [38]. These energy calculations did, however, imply that the phosphoryl transfer reaction was mixed associative/dissociative in character. Natural resonance theory calculations [39] for model associative and dissociative reactions showed that the hyperconjugation increased on approach to a dissociative transition state, and remained near constant for associative reactions [40]. Thus, for the Ras-Gap and other enzymes with a mixed mechanism, overall interactions that enhance hyperconjugation are predicted to stabilize the TS. Therefore the modulations characterized in this work could plausibly be enhancing rate as well as bond selectivity. However, we lack a detailed understanding of how the interactions might change on progression to the TS, so caution is needed in speculating about rate enhancement. Furthermore, active site interactions commonly have multiple effects which are not comparatively evaluated in the current work. Thus, the current work adds hyperconjugation as one of the plausible rationalizations of phosphoryl transfer rate enhancement, without ruling out the previously discussed electrostatic effects [5], [24], [25].

### Experimental characterization of an arginine kinase mutant

Among the diverse enzymes shown by the survey to have O_3β_ interactions, the phosphagen kinases are of particular interest. Physiologically, these enzymes buffer cellular ATP levels through a reversible transfer of the γ-phosphoryl to a guanidine substrate such as creatine or arginine [41]. The enzymes have no structural homology to those where O_3β_ interactions were previously implicated, and have not been reported to share mechanisms of catalysis. Indeed, in contrast to the dissociative mechanisms postulated for G-proteins and other enzymes with previously noted O_3β_ interactions [2], [5], [20], a more associative mechanism was suggested for phosphagen kinases [26], [27]. A high (1.2 Å) resolution crystal structure of an arginine kinase transition state analog complex added circumstantial evidence [42]. The nitrate, mimicking a transferring phosphoryl was situated between the guanidino N and nucleotide O_3β_ which were separated by 6 Å, consistent with partial axial bonding of a pentavalent P_γ_, and the angles of approach for guanidino N and O_3β_ were within a few degrees of optimal for nucleophilic attack in an early stage of the reaction, before deprotonation of the guanidino N. This suggested an active site in which enzyme interactions were optimized for a significantly associative reaction. Such contrasts to the aforementioned G proteins, together with the availability of high resolution structure, rendered arginine kinase particularly suitable as an experimental test of the predictive power of the database analysis. Below we report an experimental validation of the importance of O_3β_ interactions highlighted by the database analysis, noting that the steady state kinetics can be used to investigate the overall role of an interaction, but not detailed mechanism.

**Figure 12:**
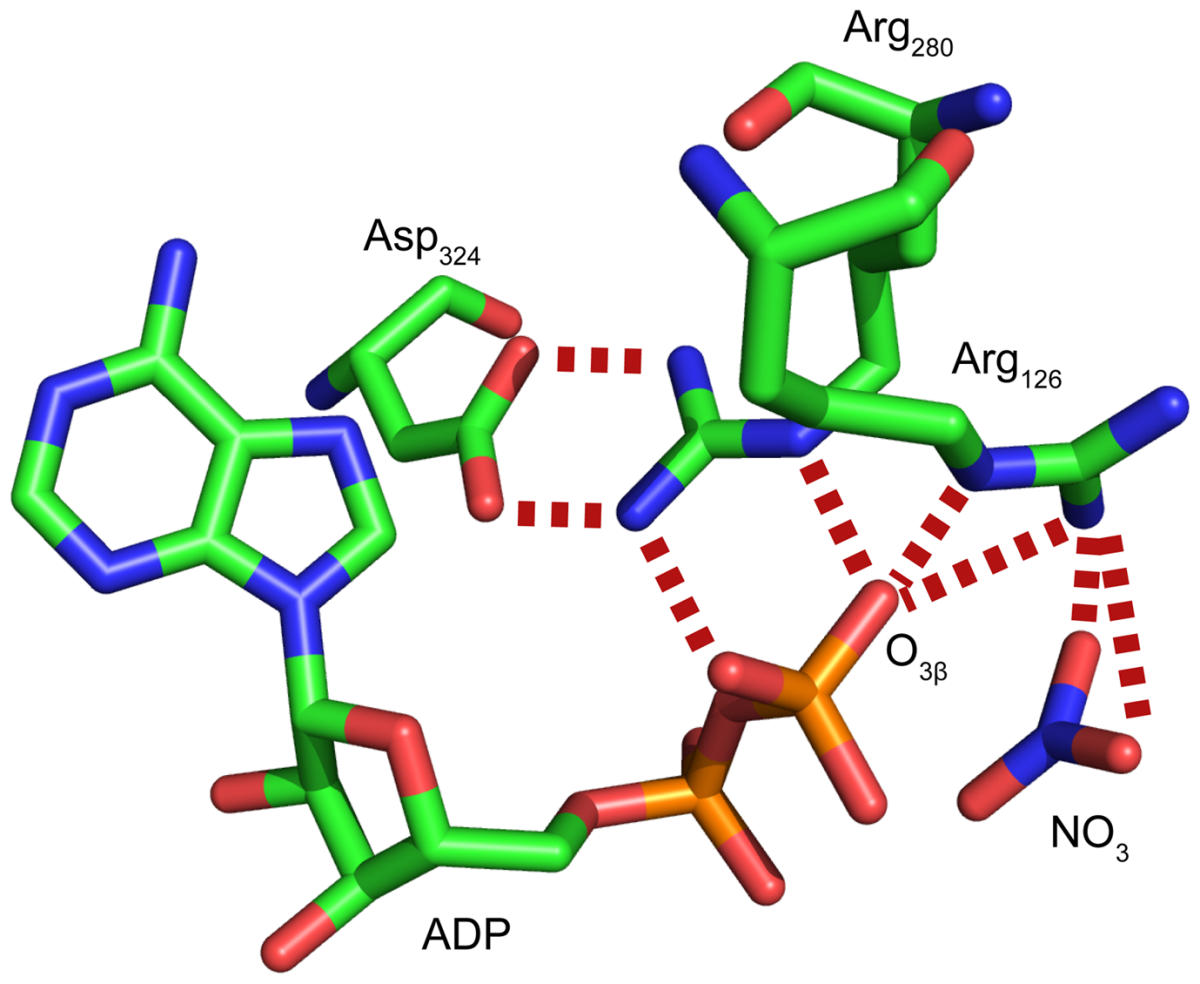
Arginine kinase active site mutation. Selected interactions of the nucleotide in the crystal structure of the transition state analog complex of Horseshoe Crab Arginine Kinase (AK, PDB ID 1M15) [48]. Carbon = green, nitrogen = dark blue, oxygen = red and phosphorus = orange. In AK, Arg_280_ contacts the O_3β_ oxygen of ADP, an α-oxygen and the Asp_324_ side chain. In ATP, the O_3β_ oxygen bridges to the γ-phosphate which is mimicked by nitrate in this transition state analog complex. Hydrogen bonds are shown with red dotted lines.

In the arginine kinase structure, the O_3β_ interacts with Arg_280_ and Arg_126_. Arg_126_ interacts with both O_3β_ and O_γ_, so Arg_280_, which also interacts with an α-nonbridging oxygen of the nucleotide substrate (and an enzyme aspartate) was chosen for mutagenesis, because it did not have potentially confounding interactions with γ-oxygens (Figure 12). Preliminary analysis showed that the activity of a R280K mutant was sufficient for steady-state kinetics analysis, but not that of a R280L mutant. Relative to the wild-type (WT), k_cat_ of R280K was decreased from 104 s^−1^ to 1 s^−1^, whereas K_M_ values for the two substrates remained within 3-fold of WT (R280K: K_M_(Arg) = 0.8 mM; K_M_(ATP) = 0.5 mM. WT K_M_(Arg) = 0.3 mM; K_M_(ATP) = 0.3 mM) [43]. The much larger impact upon rate indicates that the effect of the mutation is predominantly upon the chemical reaction as opposed to substrate binding.

The arginine kinase R280K is analogous to the R291K mutant of the homologous enzyme, creatine kinase (CK) in rabbit muscle, which yielded similar kinetics (k_cat_ = 2.2 s^−1^ and K_M_(ATP) = 1.6 mM) [44]. It was suggested that this mutation abrogated catalysis by misaligning the substrates. However, near wild-type K_M_ values argue against gross distortions of substrate-binding, although the effects of subtle modulations of alignment cannot be ruled out [45], [46]. Our database survey now highlights the analogy of the O_3β_ interaction with the corresponding ones in G-proteins and other enzymes that have been discussed above. Thus, one should consider whether there could be a holistic rationalization of O_3β_ interactions, common to phosphagen kinases, G proteins and other enzymes.

The 100-fold rate reduction of R280K is commensurate with changes on conservative mutations of arginine kinase Glu_225_ and Cys_271_ that are implicated respectively in base catalysis and electrostatic modulation of the guanidino substrate [45], [47]. Thus, the R280K mutant adds to the picture of phosphagen kinases employing several means of enhancing rate, of which the O_3β_ interactions are an important, but not dominant component. Furthermore, it is noted that the wild-type turnover is not limited by chemistry, but by protein conformational change [17]. Thus any of these mutations could be having a greater effect upon the chemistry, because we are only able to measure the part of slowing beyond the 104 s^−1^ WT turnover.

## Conclusions

Several messages can be taken from the current work. Firstly, top-down analysis of protein structure databases can reveal commonalities in the disposition of diverse active sites catalyzing analogous reactions. Secondly, O_3β_ interactions figure prominently in a wide variety of phosphoryl transferases, not just those like the G proteins previously proposed to have a dissociative mechanism. Thirdly, modulation of hyperconjugation in selective bond destabilization can be used to rationalize the disposition of phosphate active site interactions, as a potential alternative to electrostatic rationales. Finally, we are reminded that interactions cause multiple effects within substrates, and that until more quantitative analysis allows prioritization of the various effects, stereolectronics should be considered alongside more classical explanations in understanding the workings of phosphoryl transfer enzymes.

## Supporting Information

Figure S1 **Exclusion of interactions with ion-coordinants.**
(TIF)

Table S1 **Protein Data Bank structures used in the study.**
(XLSX)

Table S2 **Structure selection criteria.**
(XLSX)

Table S3 **Pairwise t-tests between structure sets, no hydrogens.**
(XLSX)

Table S4 **Pairwise t-tests between structure sets, riding hydrogens added.**
(XLSX)

Table S5 **Structures with O_3β_ nucleoside triphosphate interactions.**
(XLSX)

Table S6 **Salt bridges to nucleotide γ-oxygens.**
(XLSX)

Table S7 **Pairwise t-tests for interactions between different oxygen types.**
(XLSX)
