# Supplementary figures and images for: Common Hydrogen Bond Interactions in Diverse Phosphoryl Transfer Active Sites
Source: PLoS One. 2014 Sep 19;9(9):e108310. doi: 10.1371/journal.pone.0108310 (PMC4169622; doi:10.1371/journal.pone.0108310)

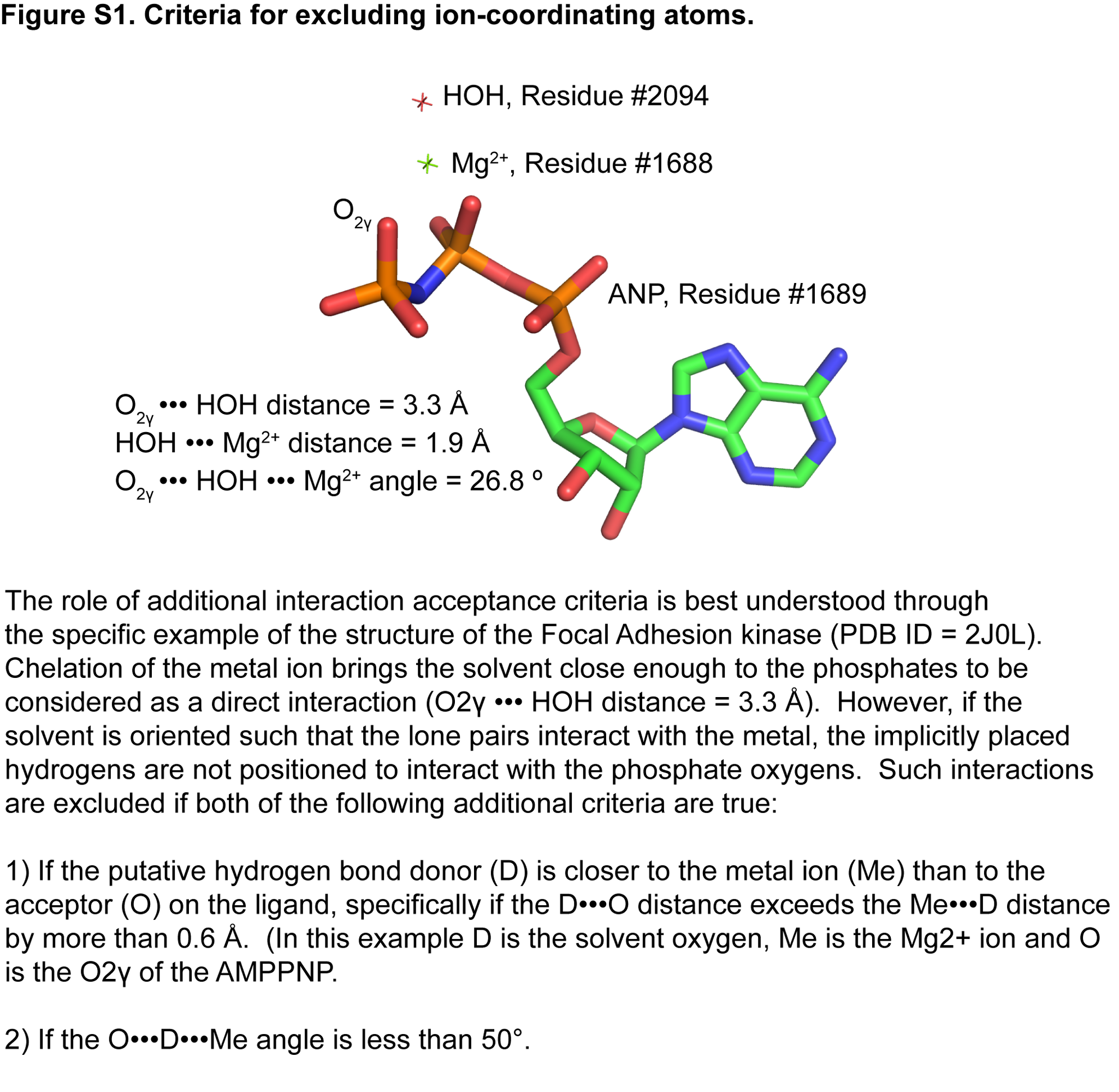

Supplement: Figure S1 — Exclusion of interactions with ion-coordinants. (TIF) [file pone.0108310.s001.tif]
